# Insulin-Mediated Activation of the L-Arginine Nitric Oxide Pathway in Man, and Its Impairment in Diabetes

**DOI:** 10.1371/journal.pone.0061840

**Published:** 2013-05-02

**Authors:** Niwanthi W. Rajapakse, Abigail L. Chong, Wei-Zheng Zhang, David M. Kaye

**Affiliations:** 1 Heart Failure Research Group, Baker IDI Heart and Diabetes Institute, Melbourne, Australia; 2 Department of Physiology, Monash University, Melbourne, Australia; 3 Department of Medicine, Monash University, Melbourne, Australia; Virgen Macarena University Hospital, Spain

## Abstract

**Aims/Hypothesis:**

Impaired L-arginine transport has been reported in cardiovascular diseases, providing a possible mechanism for reduced nitric oxide (NO) production. Given that cardiovascular diseases are also associated with insulin resistance, and insulin is known to induce vasodilation via a NO-dependent pathway, we hypothesised that abnormal insulin modulation of L-arginine transport may contribute to vascular dysfunction in diabetes.

**Methods:**

Forearm blood flow (FBF) responses to insulin and sodium nitroprusside (SNP) were measured in control and type 2 diabetic volunteers using venous occlusion plethysmography. Effects of intra-arterial insulin on the forearm veno-arterial flux of arginine and related amino acids were determined by HPLC. The effect of locally delivered insulin on arginine transport was assessed during an intra-arterial infusion of [4,5-^3^H] L-arginine.

**Results:**

In controls, intrabrachial infusion of 5 mUnits/min insulin lead to a progressive rise in FBF (p<0.001) while this was not evident in diabetics. In support of this observation, we observed a concomitant, significant increase in the flux of N-hydroxy-L-arginine (the NO precursor) in controls (baseline vs. 60 mins insulin: 16.2±12.2 vs. 33.0±13.1 nmol/100 ml tissue/min; p<0.01), whilst no increase was observed in diabetics. Moreover, insulin augmented the clearance of [^3^H]L-arginine from the forearm circulation in controls (baseline vs insulin: 123±22 vs. 150±28 ml/min; p<0.05) but not in diabetics.

**Conclusion:**

These findings suggest that insulin resistance may contribute substantially to the onset and development of cardiovascular disease in type 2 diabetics via abnormal insulin-mediated regulation of L-arginine transport.

## Introduction

Endothelial dysfunction has been associated with several disorders affecting the cardiovascular system, including type 2 diabetes, essential hypertension, hypercholesterolemia, obesity, atherosclerosis, coronary artery disease, and congestive heart failure [1]–[7]. In this context, we and others have recently demonstrated that L-arginine transport is impaired in both heart failure and hypertensive subjects with established endothelial dysfunction [8]–[10]. As the supply of L-arginine substrate to endothelial nitric oxide synthase (eNOS) is the rate limiting step in the L-arginine to nitric oxide (NO) pathway, such impairment may provide a potential mechanism for reduced NO production, and therefore endothelial dysfunction.

Insulin has been previously reported to exert vasodilator actions that are significantly attenuated when co-infused with the L-arginine analogue, L-N-mono-methyl-arginine (LNMMA), which competitively antagonises the synthesis of NO [11]. This has been interpreted to suggest that insulin increases blood flow by stimulating endothelium-derived NO production, however the precise underlying mechanisms remain unclear. Whilst several studies link insulin resistance with endothelial dysfunction, the specific nature of their relationship has yet to be elucidated. Therefore the present study sought to assess the role of insulin in L-arginine transport and endothelial function under normal conditions and in type 2 diabetes. We hypothesised that insulin facilitates L-arginine transport, thereby augmenting endothelial function and inducing a NO-dependent vasorelaxant effect.

## Methods

### Subjects

We studied 11 healthy, normotensive male volunteers and 9 type 2 diabetic males. At a screening visit, physical health was confirmed by history and physical examination, and supine blood pressure was measured in triplicate (Dinamap Critikon vital signs monitor 1849 SX). Participants were classified as normotensive if blood pressure was <130 mm Hg systolic and <80 mm Hg diastolic. Blood pressure readings were confirmed by intra-arterial blood pressure measurement during the forearm blood flow study. Healthy subjects were not on any concurrent medication or dietary supplementation (including antioxidants). Type 2 diabetic subjects continued treatment with oral hypoglycaemics and/or diet only, but refrained from taking their medications on the morning of the studies. Diabetics on insulin therapy were excluded from this study. All subjects were non-smokers, and abstained from caffeinated drinks prior to both study sessions, which were at least 3 weeks apart. Informed consent was obtained from all participants following explanation of the nature of the study. The study was approved by the Alfred Hospital Ethics Review Committee, which is constituted according to the National Health and Medical Research Council (NHMRC) of Australia guidelines. All research was performed in accordance with the Declaration of Helsinki (2004) of the World Medical Association.

### Forearm vascular and biochemical responses to intra-arterial insulin

The study commenced at 10 am after an overnight fasting period of at least 12 hours. Under local anaesthesia (lignocaine 1%, Astra, Australia), a 3 Fr catheter (Cook, Brisbane, Australia) was inserted into the brachial artery for the infusion of radiolabeled L-arginine and arterial blood sampling. A 5 Fr venous catheter (Cook, Australia) was inserted in a retrograde fashion into a deep antecubital vein for venous blood sampling, as previously described [8], [9]. After a stabilisation period of 30 minutes, resting forearm blood flow was measured and an intra-arterial infusion of tritiated [4,5-^3^H]L-arginine (ICN Radiochemicals; specific activity 98 to 106 Ci/mmol) commenced at a flow rate of 6 ml/hour, delivering 1.0 µCi/min, for 30 minutes to steady state.

Following the initiation of the [^3^H]L-arginine intra-arterial infusion, a concomitant infusion of 5 mUnits/min[12] regular human insulin (Actrapid; Novo Nordisk Pharmaceuticals, Denmark) was administered at 30 ml/hr for 60 minutes. To ensure that this dose did not affect blood glucose, we measured serial (10 minutes) blood glucose concentration using bedside analyser (Cholestech LDX Lipid analyser, Hayward, CA, USA). At the conclusion of the insulin infusion a 20 minute washout period was allowed to re-establish baseline blood flow. Graded infusions of the endothelium independent vasodilator sodium nitroprusside were then administered at 2, 4 and 8 ng/min at a flow rate of 2 mL/min. On a separate day, saline was infused at 30 ml/hr for 60 minutes to determine whether intra-arterial infusion per se altered forearm blood flow. Infusions of saline or insulin were performed in random order. Vasodilator responses were assessed by measuring forearm blood flow (FBF) using strain-gauge plethysmography (SDR Clinical Technology, Australia) as described previously [13].

### Biochemical measures

Prior to and during the insulin infusion, simultaneous arterial and deep venous blood samples were drawn and immediately transferred to ice chilled EGTA tubes and stored on ice until the completion of the study. On the same day, blood samples were centrifuged at 4°C, after which the plasma was separated and stored at −80°C. Arterial and venous plasma concentrations of arginine, N^ω^-hydroxy-L-arginine (NOHA) and asymmetric dimethylarginine (ADMA) were measured by reverse-phase liquid chromatography with timed *ortho*-pthal-dialdehyde (OPA) pre-column derivatisation, as previously described [14]. The plasma concentration of [^3^H]L-arginine was determined using ion-exchange chromatography, and an index of arginine uptake in the forearm and the rate of [^3^H]L-arginine clearance was calculated as described previously [8].

### Statistical analysis

Group data are presented as mean ± SEM. Between group comparisons were performed using Students t-test. Analysis of time and dose response relationships were analysed by ANOVA for repeated measures. A probability value <0.05 was considered to indicate statistical significance. The data were processed with the use of the software package SigmaStat for Windows 3.10 (Systat Software, Inc). SPSS Statistics 17.0 was used to perform ANCOVA (covariates age, BMI, MAP and HDL cholesterol).

## Results

### Group characteristics

Baseline characteristics of the study cohort are summarised in Table 1. Healthy volunteers had normal blood pressure, and normal fasting glucose, lipid and insulin profiles. We aimed to recruit type 2 diabetics with as few co-morbidities as possible. Not unexpectedly however, diabetic subjects were older (p<0.001), had higher blood pressure (systolic p<0.05, diastolic p<0.01 and mean arterial p<0.01), higher BMI (p<0.01) and had elevated fasting glucose levels (p = 0.001). In this cohort, diabetics also had similar triglycerides and total cholesterol levels, while they had lower HDL levels (p<0.01).

**Table 1:** Clinical characteristics of the study cohort.

	Control (n = 11)	Type 2 Diabetes (n = 9)	*p*
Age, years	27±4	54±3	<0.001
BMI, kg/m^2^	23±2	29±7	<0.01
Systolic blood pressure, mm Hg	121±3	133±4	<0.05
Diastolic blood pressure, mm Hg	6±3	76±2	<0.01
Mean arterial pressure, mm Hg	84±2	95±2	<0.01
Total cholesterol, mmol/L	4.2±0.2	4.2±0.5	ns
HDL cholesterol, mmol/L	1.4±0.1	1.0±0.1	<0.01
Triglycerides, mmol/L	1.0±0.2	1.3±0.1	ns
Plasma glucose, mmol/L	4.7±0.1	9.3±0.7	<0.001

### Vascular responses to insulin infusion and sodium nitroprusside

**Figure 1:**
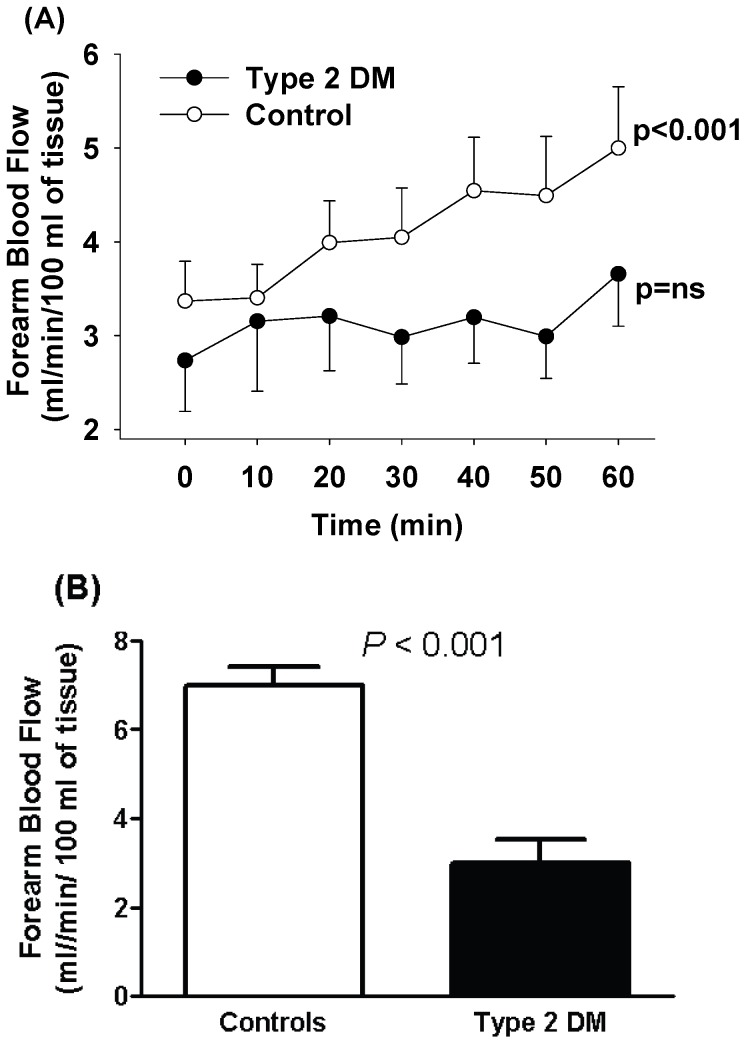
(**A**) Forearm blood flow responses to intra-arterial insulin infusion in controls (n = 11) and type 2 diabetic subjects (n = 9). Symbols and error bars represent mean±SEM. *P* values refer to 1-way RM ANOVA statistic. Type 2 DM, subjects with type 2 diabetes mellitus. (**B**) Forearm blood flow responses to intra-arterial insulin averaged over a 60 min period in controls (n = 11) and type 2 diabetic subjects (n = 9). Error bars are SEM. *P* value is the outcome of an ANCOVA.

At baseline, FBF tended to be slightly lower in diabetics as compared to controls (control vs diabetics, 3.4±0.4 vs 2.7±0.5 mL.min^−1^.100 mL^−1^), although the difference was not statistically significant. In the time control studies, infusion of saline alone did not alter forearm blood flow over the 60 minute infusion period (data not shown). Infusion of insulin at 5 mUnits/min over a 60 minute period lead to a significant increase in FBF in healthy controls from 3.4±0.4 to 5.0±0.7 mL.min^−1^.100 mL^−1^ (p<0.001), whilst in diabetics there was a small although non-significant rise from 2.7±0.5 to 3.6±0.6 mL.min^−1^.100 mL^−1^ (p = ns) (Figure 1A). The increase in forearm blood flow during insulin infusion was 57% less in diabetic subjects compared to controls after adjusting for covariates age, BMI, MAP and HDL-cholesterol (Figure 1B). No significant changes in blood pressure, heart rate or peripheral plasma glucose were observed during insulin administration in either group.

**Figure 2:**
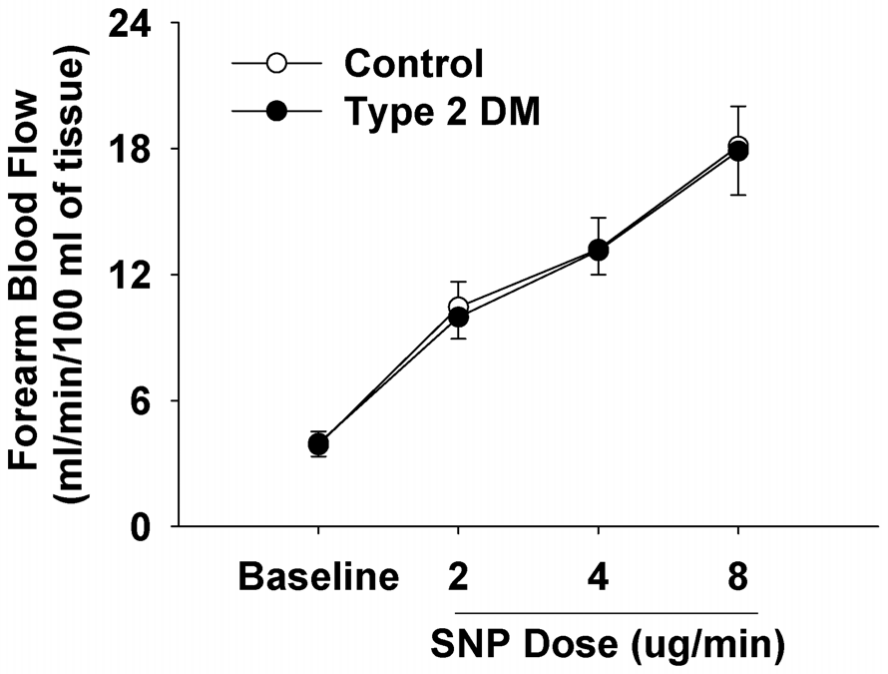
Forearm blood flow responses to intra-arterial sodium nitroprusside infusion in controls (n = 11) and type 2 diabetic subjects (n = 9). Symbols and error bars represent mean±SEM. Type 2 DM, subjects with type 2 diabetes mellitus; SNP, sodium nitroprusside.

Intra-arterial infusion of sodium nitroprusside led to a dose-dependent increase in FBF in both controls and diabetics subjects, and the increment was of a similar magnitude in both groups (Figure 2). In controls subjects FBF increased from 3.8±0.6 to 18.1±1.9 mL.min^−1^.100 mL^−1^ (p<0.001) in response to 8 ng/min SNP, whilst in diabetics it increased from 3.9±0.6 to 17.8±2.1 mL.min^−1^.100 mL^−1^ (p<0.001).

### Effect of insulin on Arginine Transport and Metabolism

**Figure 3:**
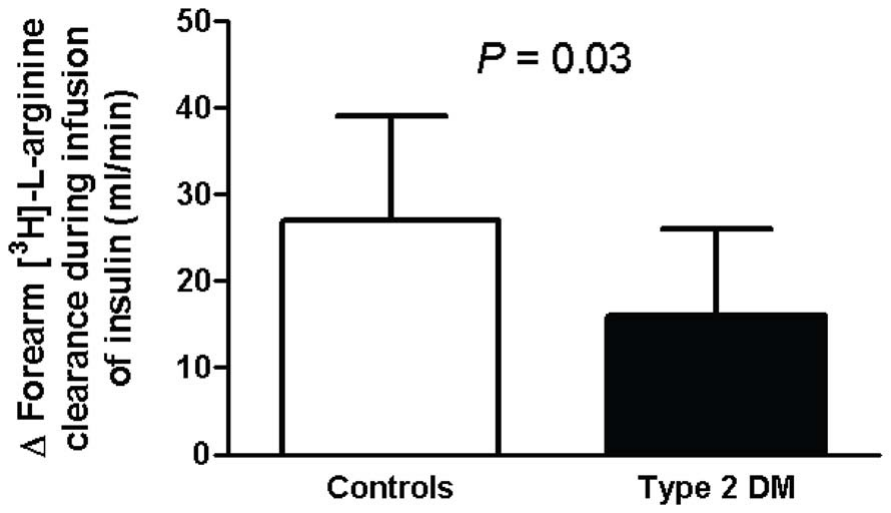
Effects of intra-arterial infusion of insulin on forearm ^3^[H]-L-arginine clearance in controls (n = 11) and type 2 diabetic subjects (n = 9). Open and closed bars represent absolute changes in forearm ^3^[H]-L-arginine clearance (ml/min) during infusion of insulin compared to respective baseline levels in controls and type 2 diabetic subjects, respectively. Error bars are SEM. *P* value was derived from ANCOVA.

Insulin augmented [^3^H]L-arginine clearance by 22% in control subjects (baseline vs insulin: 123±22 vs. 150±28 ml/min; p<0.05) while only a 10% rise was observed in diabetic subjects (baseline vs insulin: 160±24 vs 175±32 ml/min; p = ns) across the forearm. The increase in [^3^H]L-arginine clearance during insulin infusion was 41% less in diabetic subjects compared to controls after adjusting for covariates age, BMI, MAP and HDL-cholesterol (Figure 3). There was no significant difference in the forearm [^3^H]L-arginine clearance between the two groups at baseline.

At baseline, there were no detectable differences in the arterial plasma concentrations of L-arginine, NOHA or ADMA between control and diabetic subjects (Table 2). In both study groups, insulin infusion had little effect on baseline plasma concentrations of L-arginine, NOHA or ADMA (data not shown). The baseline veno-arterial fluxes of L-arginine, NOHA and ADMA were also similar across the two groups (Table 2). Consistent with the uptake of L-arginine across the forearm, a net negative arginine veno-arterial was observed in both study groups, which is also in keeping with the above [^3^H]L-arginine clearance data.

**Table 2:** Biochemical characteristics of the study cohort.

	Control	Type 2 Diabetes
**Plasma Arterial Concentration**		
L-Arginine ( µmol/L)	124.3±20.2	114.5±10.9
NOHA ( µmol/L)	9.0±1.5	13.2±1.3
ADMA ( µmol/L)	1.9±0.2	1.5±0.5
**Flux (nmol.100 mL tissue/min)**		
L-Arginine	−77.3±36.1	−56.8±40.5
NOHA	16.2±12.2	13.7±4.7
ADMA	−1.9±1.6	1.1±.6

No significant differences between groups. NOHA (N^ω^-hydroxy-L-arginine),

ADMA (asymmetric dimethylarginine) (n = 9–11 per group).

**Figure 4:**
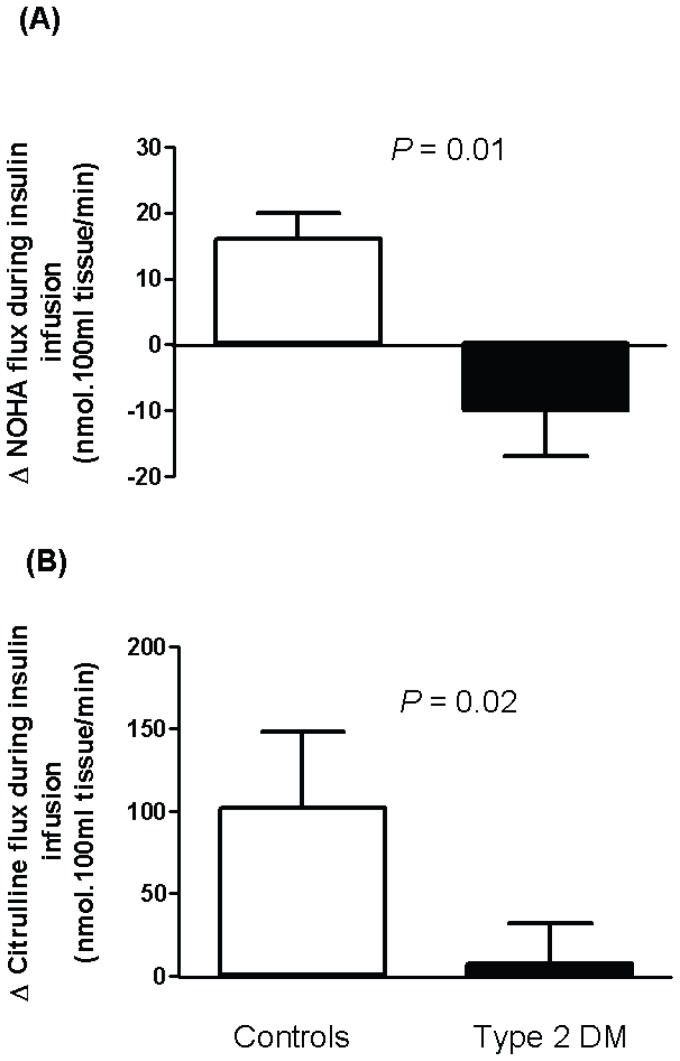
Effects of intra-arterial infusion of insulin on forearm flux of NOHA and citrulline. Bar graphs represent the absolute changes in forearm flux of (**A**) NOHA and (**B**) citrulline during infusion of insulin compared to respective baseline levels in controls (n = 11) and type 2 diabetic subjects (n = 9). Error bars are SEM. *P* values were derived from ANCOVA. Type 2 DM, subjects with type 2 diabetes mellitus, NOHA, N^ω^-hydroxy-L-arginine.

In response to insulin infusion, there was a significant increase in the flux of the NO intermediate, NOHA in control subjects (p<0.01), whilst in diabetics this tended to fall (p = 0.08). In healthy subjects there was also a trend towards increased L-citrulline flux (p = 0.07), also consistent with activation of the L-arginine:NO pathway. After adjusting for covariates age, BMI, MAP and HDL-cholesterol, the increase in the flux of NOHA and citrulline during insulin infusion was 162 and 93% less respectively in diabetic subjects compared to controls (Figure 4). Net fluxes of L-arginine were not significantly affected by insulin as reflected by the total veno-arterial flux. However we did not incorporate the radiotracer data and did not attempt to mathematically model the arginine flux. We also acknowledge that these measures may have been confounded by local recycling of arginine. ADMA flux was also not appreciably changed by insulin in either group (data not shown).

## Discussion

This is the first *in vivo* biochemical study in humans to provide evidence that the vasodilatory effects of insulin are associated with activation of the L-arginine-NO pathway and this appears to be impaired in type 2 diabetes. Endothelial dysfunction is a key feature of many cardiovascular diseases and their associated risk factor states. The control of vasomotor tone by the endothelium is critically regulated by the bioavailability of NO, which in turn reflects the balance of NO biosynthesis and degradation. In this context, studies in cell culture systems demonstrate that the extracellular availability of the NO precursor, L-arginine, is a key determinant of NO production [15]. At the clinical level, we have demonstrated that L-arginine transport is impaired in hypertension and heart failure, both diseases in which endothelial dysfunction has been established [8], [9]. Impaired L-arginine transport may therefore provide a possible mechanism for reduced NO production; however, the underlying factors have yet to be elucidated. Given that insulin is a well known vasodilator whose actions are mediated via an endothelium-dependent pathway [10], [16], [17] and that insulin resistance has been associated with both endothelial dysfunction and reduced NO bioavailability [18], we sought to assess the role of insulin in L-arginine transport and endothelial function in man under normal physiological conditions and in type 2 diabetes.

In the current study we observed that the local intra-arterial infusion of insulin induced a vasodilatory effect as evidenced by a gradual, significant increase in forearm blood flow in healthy volunteers while only a small non-significant rise was seen in type II diabetics. Importantly, this impaired insulin dependent vasodilatation in diabetics compared to controls was present even after adjusting for potentially confounding factors; age, BMI, MAP and HDL-cholesterol. Thus, this observation is in agreement with some previous reports [19], [20], but in contrast to others [21]–[24]. It is possible that differences in dose and infusion time may explain the discrepancies in the findings. Our data are consistent with previous data which indicate that acetylcholine dependent endothelial vasodilation is impaired in type 2 diabetes [25]. In conjunction with the documentation of the vasodilator response to insulin, we demonstrated activation of the NO synthetic pathway, thereby providing a novel insight into the potential mechanism for the endothelial actions of insulin.

There is conflicting evidence as to whether the effects of insulin are mediated at the level of the endothelium or whether other systemic mechanisms are involved (reviewed in [26]). In our study, blood pressure, heart rate and peripheral plasma glucose did not change significantly during drug administration, suggesting that the insulin-induced vasodilation was a result of a direct interaction with the vasculature rather than that of systemic influence. We demonstrated that insulin increased the release of the NO intermediate, NOHA, potentially by activating the transport of L-arginine in the forearm, suggesting that the effects of insulin were mediated, at least partially, via an endothelium-dependent pathway. The mechanism by which insulin might alter arginine transport was not addressed in this study. In this context, it has previously been shown that insulin increases L-arginine transport in cultured endothelial cells by increasing the expression of cationic amino acid transporter-1 (CAT-1), which is the predominant L-arginine transporter expressed in endothelial cells [27].

Alternatively, it is possible that the insulin receptor (IR) may influence the principal L-arginine transporter, CAT-1, via other direct or indirect mechanisms. The insulin receptor substrate (IRS) protein is activated following the IR binding to the ligand with subsequent phosphorylation of IRS by IR tyrosine kinase. Once activated, IRS proteins are able to stimulate the phosphoinositide 3-kinase (PI3K) pathway, which results in the activation of PKB/Akt [28]. In cultured endothelial cells, stimulation of PKB/Akt by insulin has been shown to result in phosphorylation of the Ser1177 residue on eNOS [29], [30], which increases eNOS activity by several-fold [28], [31]. Thus, insulin may cause vasorelaxation via the activation of eNOS, which could also explain the observed increase in NOHA flux, possibly also stimulating L-arginine transport via an uncertain mechanism [15]. It is also of note that the IR has been demonstrated to be co-localised with the caveolins within plasmalemmal caveolae in rat adipoctyes [32]. Caveolin-1 and -3 have been shown to enhance insulin-stimulated tyrosine phosphorylation of IR in a dose-dependent manner by directly interacting with a specific caveolin-binding motif in the IR [33]. Caveolins are also known to co-localise with both eNOS and CAT-1 [34].

Our findings have direct relevance to understanding the pathogenesis of the association between endothelial dysfunction and insulin resistance. Insulin resistance is characterised by an impaired response to the actions of insulin, which results in a compensatory increase in insulin (hyperinsulinemia) in an attempt to maintain normal blood glucose levels [35]. Insulin resistance has been found in type 2 diabetes, hypertension, dyslipidemia, obesity, chronic heart failure and in atherosclerosis [36]–[39], and has been suggested to be an independent risk factor of cardiovascular disease [40]. Endothelial dysfunction has been associated with insulin resistance in patients with coronary artery disease, and has been detected in normotensive individuals with a familial history of insulin resistance and type 2 diabetes [41], [42]. In addition, insulin sensitivity has been correlated with basal endothelial NO production in healthy males [18]. Therefore, in light of the above, it is possible that insulin resistance may contribute substantially to the onset and development of cardiovascular disease associated with insulin resistance through abnormal insulin-mediated regulation of L-arginine transport. As a corollary of our findings, new approaches to the modulation of vascular tone in insulin resistant states may require direct manipulation of the endothelial arginine-NO pathway.

Our study is consistent with the concept that insulin induces vasodilation by activation of the endothelial L-arginine NO pathway, supported particularly by the observation insulin caused a rise in the release of NOHA in healthy subjects. Others have proposed that the vascular actions may be in part explained by the recruitment of capillaries rather than vasodilation of active resistance vessels [12], [43]. This hypothesis may explain, in part the effects of insulin on skeletal muscle glucose uptake although this remains controversial. Nevertheless, it is also acknowledged that capillary recruitment is probably NO dependent, which is consistent with our findings. In previous studies [9], we showed that an increase in blood flow per se, for example that mediated by the endothelium independent vasodilator sodium nitroprusside does not alter [^3^H]L-arginine clearance, therefore suggesting the effect of insulin was specifically due to an action on L-arginine transport. However, from the present in vivo study, it is not possible to specifically identify endothelial cells as the major site of arginine clearance.

In the present study we did not observe a significant change in the flux of L-arginine or citrulline (although a trend was seen in the normal subjects), despite the increase in NOHA flux. We did not however attempt to apply any mathematical models to more formally investigate the local kinetics of arginine. Importantly, it should be noted that the plasma L-arginine and citrulline concentrations are larger than the plasma concentration of NOHA by approximately one-and-a-half to two orders of magnitude. Accordingly, on a direct molar basis it would be unlikely that a significant veno-arterial concentration gradient could be detected for unlabelled arginine or citrulline.

In conclusion, our study provides a novel, in vivo, mechanistic insight into the vascular actions of insulin. Moreover, the data suggest a potential basis for the association between type II diabetes, insulin resistance and endothelial dysfunction. The present study warrants further research into the contribution of insulin resistance to impaired L-arginine transport and endothelial dysfunction in other insulin resistant states, such as hypertension and congestive heart failure, which are also characterised by endothelial dysfunction and impaired L-arginine transport.
